# Identifying the non-governmental organizations' activities and challenges in response to the COVID-19 pandemic in Iran

**DOI:** 10.1186/s12889-022-13080-5

**Published:** 2022-04-10

**Authors:** Azadeh Sayarifard, Maryam Nazari, Fatemeh Rajabi, Laleh Ghadirian, Haniye Sadat Sajadi

**Affiliations:** 1 Community Based Participatory Research Center, Iranian Institute for Reduction of High-Risk Behaviors, Tehran University of Medical Sciences, Tehran, Iran; 2 Department of Healthcare Management and Economics, School of Public Health, Tehran University of Medical Sciences, Tehran, Iran; 3 University Research and Development Center, Tehran University of Medical Sciences, Tehran, Iran; 4 Knowledge Utilization Research Center, Tehran University of Medical Sciences, Tehran, Iran

**Keywords:** Health system, Public participation, Non-governmental organizations, Covid-19, Iran

## Abstract

**Background:**

The spread and severity of the COVID-19 pandemic have been to such an extent that to shape an effective, collective response, governments need the participation of society and the cooperation of a wide range of civil society organizations and institutions. The objective of this study was to identify the activities of non-governmental organizations (NGOs) in response to the covid-19 pandemic in Iran and the challenges they confronted.

**Methods:**

We conducted a qualitative analysis based on twenty-two individual, virtual, and semi-structured interviews. Interviewees were selected through purposeful and snowball sampling. All interviews were performed with active health-related NGO representatives willing to participate in the study and continued until data saturation. Data analysis was performed using qualitative content analysis.

**Results:**

The activities and challenges raised by NGO representatives were identified in 6 main categories, including the need for the participation of NGOs in the fight against pandemics, response to the COVID-19 crisis in the society, challenges in providing services to the target group in the COVID-19 crisis, NGOs challenges in interacting with governmental and non-governmental institutions, information sources used by NGOs in the COVID-19 crisis and strategies to support NGOs in their efforts.

**Conclusion:**

Considering the crucial challenges for their participation, such as the NGO access to the target groups, lack of communication network, and constructive interaction between government institutions and the NGOs, it is recommended to increase the capacity of these institutions and intervene to establish a constructive and long-term relationship with the government.

## Background

The new Coronavirus disease emerged in December 2019 in Wuhan, China, spreading worldwide [1]. The spread and severity of the COVID-19 pandemic have been to such an extent that governments need the participation of society and the cooperation of a wide range of civil society organizations and institutions [2] to shape an effective, collective response because their capacity to control crisis and restore the situation to normal is limited [3–6]. Non-governmental organizations (NGOs) constitute a range of organizations that are partially or entirely independent of the government and have humanitarian or cooperative rather than commercial goals. NGOs have gained more power in the field of health over time [7].

Many scholars reported that organized and harmonized activities of NGOs can lead to a better and more effective response to the crises in the country [3, 8]. NGOs have a closer connection with the communities, benefit from more flexible processes, and require less paperwork than governmental institutions to respond more quickly to crises [9]. Rahma's report on the activity of the NGOs during the outbreak of bird flu in Indonesia noted that the government alone could not disseminate the large volume of information to people living on different islands to fight the bird flu in 2006. Therefore, it took advantage of the capacity of the NGOs and their support in this regard, especially in the field of NGO staff and network development. The report recommended that these organizations be equipped and supported in times of crisis to address the community's need, as the NGOs can bridge the gap between government and grassroots levels [10]. In Wong's 2002 study, the participation of the NGOs in response to acute and general crises such as SARS was also seen as a sign of complementarity between civil society and the state [11]. The experience of several countries in response to COVID-19 [12–15] also showed that NGOs play a vital role when national governments cannot alone manage to fulfill the needs of the population. While restrictive policies toward society significantly limited the role of NGOs, they were still actively involved in emergency service, funding, volunteering, mutual aid, in-kind donations, and policy advocacy in several countries. Studies have also reported that NGOs and community-based organizations have been a vital determinant of the effectiveness of local restraint strategies [16].

Given that the COVID-19 pandemic has not yet been controlled, there is an unprecedented need for all stakeholders to play their role in restraining this widespread disease. In addition, considering that the condition is expected to have many short-term and long-term effects on a large number of highly vulnerable individuals, the NGOs must be strengthened for timely response to the COVID-19 crises. However, it is unclear how NGOs should be optimized, mobilized, and exploited in response to COVID-19 and the existing mechanisms [2]. Also, NGOs will not be immune to administrative constraints due to the pandemic, which will hamper the continuity of their activities and affect their capacity to provide services to vulnerable families [17]. Hence, more investigations are required to analyze the response of NGOs during COVID-19 in different contexts and identify their challenges. These investigations have lessons to improve NGOs' performance in the crisis. The NGOs are the closest institutions to the public within a society, so empowering them to participate in crisis response can turn these institutions into an auxiliary arm of the government in crisis management [18]. This study aimed to identify the activities of NGOs in response to the COVID-19 pandemic in Iran and the challenges they confronted.

## NGOs in Iran and their role in coping with COVID-19

Like many countries, NGOs in Iran are an integral part of civil society, concerned with managing a complex and diverse range of activities. The exact number of Iranian NGOs and their working activities are unclear [19]. However, the growing number of NGOs and the scope of operations they have taken up, as articles 95, 97, 104, 140, and 163 of the Iranian Government's Fourth Development Plan, stress the participation of NGOs in development plans [20, 21]. The Iranian NGOs are involved in different area, including health, women, the environment, children's wellbeing, and training. Their work can be divided into the following categories: informative, educational, operational consultation, small projects, supportive, production, and training [22]. Studies reported Iranian NGOs to face many challenges. NGO registration system, interaction with other NGOs, private sectors and government, and internationalization and financial crisis are the most challenging issues facing the NGOs. Despite these challenges, the NGOs helped Iran to cope with COVID-19 [23].

Following the ongoing COVID-19, Iran's efforts have begun to tackle. The contextual-based policies have been formulated to combat the COVID-19 outbreak, including risk assessment and effectiveness of preparedness and response measures; production and logistics of medicines and medical equipment; effective disease surveillance and response measures; establishing national campaign; and vulnerability mapping. Many operations were carried out by popular groups or NGOs concerning community engagement. Some of the examples are participation in disinfecting the cities; voluntary contributing of the neighborhood community in the production of some personal protective equipment, accompanying screening groups or non-governmental entities for the home visits, and coordination in collection and distribution of financial support and food packages to the poorest members of the community [24, 25]. NGOs can also raise public awareness, alter social policies, and provide services and facilities to vulnerable groups in dealing with crises [26]. Despite these initiatives, it was reported that an insufficient whole-government, whole-society approach in managing the outbreak, is one of the biggest challenges in policymaking to combat COVID-19 [27].

## Methods

This study was a qualitative study to identify the activities of NGOs in response to the COVID-19 pandemic and the challenges they had. Data were collected through 22 interviews with representatives of health-related NGOs. According to the classification done in the Social Participation Directorate of the Islamic Republic of Iran's Ministry of the Interior; 2020, the field of activity of NGOs in Iran is different. Some areas of activity of NGOs in Iran include social issues, culture and art, development, environment, and health. In this study, the target group was NGOs in health. The field of activity of the studied NGOs included prevention of social harms, women's, children's, and adolescents' health, support for the people with disabilities, support, care, and treatment for certain diseases such as cancer, Hemophilia, AIDS, diabetes, etc. NGOs are nonprofit organizations that do not hire employees, are mainly involved in technical work and service delivery, and are independent of the government or any political or religious program. While several NGOs agree with this definition, in practice, some may not. For example, some NGOs have paid personnel positions, and some are affiliated with government agencies [18]. In our study, the NGOs were not dependent on the government.

Purposeful and snowball sampling were used to select these participants. Having a history of working in the NGO for at least five years, being responsible and knowledgeable about the issues of the NGO (preferably being as NGO manager), and being willing to participate in the study and share the experiences were considered our inclusion criteria.

The interviews were individual, virtual (in the Skype), and semi-structured. The average interview time was 60 min. Interviews were conducted between February 2021 and April 2021. Due to the restrictions of the COVID-19 pandemic, the virtual method was selected for the interviews. The interview time was set to the convenience of the participants and prior arrangement. After initial coordination, the notification forms were submitted to the participants by e-mail or fax. The informed consent of the participants was obtained to respect interview ethics. In each interview, the interviewers observed the initial protocol that included mentioning the title of the project and the need to implement it, obtaining permission to record the interview with emphasis on confidentiality, declaring freedom to withdraw from the study at all stages, and sending final results to the participants if desired. All interviews were recorded. The note-taking method was also used during the interview. The interviews continued until the required results were saturated in the opinion of the researchers. Interviewees were asked about the activities performed by NGOs in response to the Covid-19 epidemic, the problems they encountered, the impact of the Covid-19 epidemic on their activities and service delivery of NGOs, and the strategies and resources that help NGOs to play a more effective role in crises.

Since all participants spoke in Persian, the interview guide was prepared in Persian. Five experts confirmed the content validity of the guide. The practicality and comprehensibility of the questions were tested through two pilot interviews, and the necessary modifications were made. Data analysis was performed using qualitative content analysis. For this purpose, the interviews were transcribed accurately. Each interview transcript was compared to the notes taken during the interview for the relevance of the spoken and non-spoken material. Participants' words denoting a semantic unit were extracted as code and categorized into primary and sub-categories. It is worth noting that all stages of interview and analysis were performed in the Persian language. Codes and quotes were translated into English when preparing the manuscript.

For reflexivity, the researchers avoided their beliefs and personal perceptions in the interpretations. For bracketing, while dynamic interaction with the participant, they avoided leading or directing the conversations. Through the member check method (participant or respondent validation) [28], the extracted codes were randomly selected and returned to the participants to ensure the consistency of the codes with the participants' experiences. Finally, the interviews and codes were reviewed for peer debriefing by experts.

## Results

In this study, 22 NGO managers from different provinces (including Tehran, Alborz, Gilan, Kermanshah, Khorasan Razavi, Khuzestan, Lorestan, and East Azarbaijan provinces) were interviewed. Participants included eight men and 14 women, all aged 35 to 60 years, with an average work experience of 12 years. Participants' education status varied from high school diploma to professional doctorate. More details of our participants' characteristics are presented in Table 1.

**Table 1:** Respondent characteristics

Variable	Frequency (*N* = 22)
**Gender**	
Female	14
Male	8
**Age**	
35–45	11
46–55	8
> 56	3
**Education level**	
Diploma	1
Bachelor	5
Master	11
Doctorate	5
**Work experience in NGO**	
3–5	3
5–10	7
> 10	12

Three hundred eighty-eight meaningful codes were extracted from the present study, classified into six main categories and 46 sub-categories. The main categories include the need for the participation of NGOs in the fight against pandemics, response to the COVID-19 crisis in the society, challenges in providing services to the target group in the COVID-19 crisis, NGOs challenges in interacting with governmental and non-governmental institutions, information sources used by NGOs in the COVID-19 crisis and strategies to support NGOs in their efforts. Details of the corresponding sub-categories are provided in Table 2.

**Table 2:** Main and sub-categories obtained from qualitative data analysis

Main categories	Sub-categories
The need for NGO participation in the fight against pandemics	The potential to face the crisis
	Alleviating the governments' burden
	Ability to gain public trust
	Awareness of community needs
COVID-19 crisis response in the community	Identification of those in need
	Provision of livelihood facilities
	Income generation and job creation
	Providing services in the field of health and disease prevention
	Build trust for vaccination
	Screening and care for families with affected or missing members
	Provision of psychological services
	Raising awareness and provision of training services
	Establishment of dynamic interaction and close collaboration with other institutions
	Hospital and clinical staff support
	Demanding and advocacy
	Change of routine services provision methods
Challenges of service provision to the target population in COVID-19 crisis	The unpredictable nature of the crisis and lack of preparedness to fight it
	Increasing vulnerability and need of the society
	Disruption in the provision of routine services in person
	Threat to the health and wellbeing of NGOs staff
	Ceasing to support from Charities
	The unfavorable technology infrastructure of the country
	Inefficiency in financial resource management
NGO challenges in interactions with government	The negative attitude of the parties to each other
	Irrational expectations of the NGOs from the government
	Non-recognition of the freedom of action of the NGOs
	Unawareness of the parties of each other's roles, plans, and capacities
	Shortcomings in the formulation and implementation of laws
	Disorders in current administrative processes
	Lack of support from the governing administrations
NGO information sources in COVID-19 crisis	Ministry of Health and related organizations
	Municipality
	Social Welfare Organization
	Peers
	National media
	National Networks and Committees
NGO support strategies in the pandemic efforts	Updated dissemination of information
	Planning and leading efforts
	Recognition of the rights, status, and role of the NGOs
	Technical and educational support
	Physical, human, and financial support
	Promotion of a culture of social participation
	Facilitation of the NGO participation process in decision making

### The need for NGO participation in the fight against pandemics

This category includes the potential to face the crisis, alleviate the governments' burden, gain public trust and awareness of community needs. NGOs can help and complement governments in the fight against epidemics by having components such as the potential to deal with crises, the ability to gain public trust, and awareness of the needs of society. According to one of the participants, the government's benefit from the presence of the NGOs will lead to success in the fight against the crisis.

*"… Crisis management in all countries, of course, in a community-based way can be more successful. The potential capacity of NGOs can be effective in reducing social crises because NGOs can play an important role by encouraging people to participate." (P2).*

*"One of the ways to ensure people's participation in crisis control, which is incidentally defined as global, is for people to participate in government decisions through their organizations. If we want to talk to the guilds, they have a union, and if we want to talk to the Chamber of Commerce, they have a representative. In the social sphere, representatives are non-governmental organizations" (P6).*

The participation of NGOs alleviates the heavy burden on the government during the crisis.

*"… It has been an issue in Iran since ancient times that people do not wait for the government in crises and rush to help each other. …The NGOs can provide support and assistance to the government and the National CORONA headquarters because they are much more agile than the government body and do not have government bureaucracy." (P2).*

NGOs can encourage public participation and build trust in society, which is the basis for the effectiveness of government interventions. On the other hand, Some NGOs are so well-known and trusted by society that most public aid is sent to them.

*"There are several NGOs in the country that are more well-known, and many donors know them and help them because they trust them …" (P5).*

*"By establishing a deep connection with the people and recognizing their real needs, the NGOs can gain the trust of the society …" (P2).*

*"The government needs mediators such as NGOs, national and charismatic personalities, artists and athletes to gain public trust…" (P6).*

Other experts believe that the NGOs have information about the needs of society that can be beneficial to the government.

*"NGOs can help the government and the National Corona headquarters in making decisions because they are from the heart of the people and are aware of the needs and conditions of the people…" (P2).*

*"Our information can be made available to the Corona headquarters that, for example, the people of this region have such priority needs, what should we do now?" (P8).*

### COVID-19 crisis response in the community

In response to the pandemic, the authorities provided a wide range of activities, including identification of those in need, livelihood facilities, income generation, and job creation, health and disease prevention services, building trust for vaccination, screening, and care for families with affected or missing members, provision of psychological services, awareness-raising and training services, dynamic interaction and close collaboration with other institutions, hospital and clinical staff support and demanding and advocacy. Also, due to the circumstances, the NGOs were forced to change how they provided their routine services. Identifying those in need was one of the NGO measures to prioritize vulnerable groups and meet their most basic needs.

*"Finding the needs, which is one of the main activities of some organizations, should be done in times of crisis, because basically in times of crisis, the vulnerable are more affected by both the crisis and the actions of the government, such as quarantine… (P4)."*

*During the pandemic, the NGOs took measures for public welfare through direct provision of livelihood facilities such as food baskets and direct financial aid. "during the pandemic, we gave about 100 baskets, which included chicken, pasta, rice, soy, tomato paste, and oil …" (P12);*

*"If someone needed treatment or a housing deposit, we would help in cash …" (P11).*

During the COVID-19 crisis, some NGOs also provided job opportunities and income generation. *"Approximately twenty recovered people set up a sewing workshop using UN training and produced 1,000 to 2,000 masks a day …" (P8).*

*The measures were the supply, production, and distribution of health items for COVID-19 prevention. "On a routine basis, we allocate quotas of masks and disinfectants, especially to families in financial difficulty …" (P22).*

NGOs can also be influential in vaccination against the disease and advocating for it due to the trust and broad access to the community.

*"We serve in the suburbs. In these areas, in the evenings, men gather in front of coffee shops, and women gather at the door of their houses and the information dissemination network is robust, it is enough to vaccinate or give a mask to one person, tomorrow 50 people will come in with the request to receive masks or vaccination… " (P5).*

NGOs were also involved in patient identification and referral.

*"We set up a 16-h center. Our facilitators, health ambassadors, and staff screened people three times. We would report suspicious cases to the hospital or tell them to stay home and be quarantined, and we would train those around them to come back if they had any symptoms. We performed PCR at the test center, and positive cases were reported …" (P5).*

With the increase of domestic tensions and psychological problems, addressing needs and providing psychological support and services was also considered.

*"We would discuss psychological issues in groups. If someone had a problem, they would raise it, and the others would try to support it under the supervision of our psychologist … The domestic violence case reports were very high, and the experts provided guidance…… In case of emergency, the necessary contact numbers of the welfare organization and the police were provided to the injured person. "(P22);*

*"During infection, we would establish telephone communication with the patients and their families, and we would disseminate messages of hope…" (P12).*

Through face-to-face and virtual training, prevention measures for the clients and staff were provided during the pandemic.

*"Information and training were provided by publishing informative posters, campaigns, webinars, and live Instagram training videos with the help of academics. These videos were about observing health protocols, quarantine, behavior change and coexistence with CORONA and resilience…" (P4).*

*Establishing dynamic interaction and cooperation with other institutions was an important step. "As the most specialized organization in the field of disasters in the country, we established coordination between the active NGOs and the National CORONA Headquarters to prevent parallel work…"* (P4).

Some NGOs made consumable items to the hospitals and repaired health centers.

*"Early in CORONA pandemic, we sent masks and shields worth thirty or forty million Tomans to Lorestan because the hospitals were out of it. Now we have a contract to repair a health house in Lorestan …" (P22).*

The NGOs' advocacy measures included transferring expectations to the government, immunizing vulnerable groups, and consulting with organizations and donors to take advantage of available opportunities.

*"There were several disabled people with special diseases, and we made communications with the county health department for the flu vaccine and submitted a list of names and national codes …" (P11).*

*"We made demands with the municipal officials. We had a council. We had a meeting with the governor and welfare. We demanded that we need a law to protect the disabled during the epidemic …" (P6).*

*"… After some time since the CORONA crisis, NGOs have just opened their eyes to the fact that what is their duty in the corona crisis is to follow the demands of the people from the government …(P6).*

*"Well, we followed up with many organizations to find out what to do, what support you provide, and what help you provide in the preparation of materials … (P23).*

*"…They also have a role to play in conveying the expectations of the society to the government, which should have so-called demanding … Pursuing the implementation of overdue laws, pursuing the immunization of vulnerable groups against the flu, seeking support and consulting with organizations and donors to providing health items and existing infrastructure and identifying and encouraging donors to participate … " (P1).*

Following the outbreak, the NGOs were required to change their routine service provision, including virtual and telephone, and in-person services according to health protocols, and extend their operation time.

*"We provided services through virtual or telephone networks that had a wider coverage …" (P2); "We held an individual consultation or a class of ten to twenty in a large space and at a great distance …" (P1).*

### Challenges of service provision to the target population in COVID-19 crisis

The unpredictable nature of the crisis and lack of preparedness to fight it, increasing the vulnerability and need of the society, disruption of routine in-person services, Threat to the health and wellbeing of NGOs staff, ceasing to support from Charities, the unfavorable environment of the country's technology infrastructure and inefficiency in the management of financial resources were some of the challenges faced by the NGOs in providing essential services during COVID-19 crisis. The ignorance of the disease, the uncertainty of the time out of the crisis, and the lack of planning were problems that were conceptually related to the nature of the crisis.

*"So far, I do not have close experience of the struggle of Iranian NGOs against the epidemic. I think we never have close experience of NGOs …" (P6).*

*"The state of Corona and the time of the end of this biological war on Earth are unclear …" (P8).*

*"We were shocked and did not think we should store materials such as masks and disinfectants…." (P8).*

Rising inflation, prices, poverty, unemployment, family disputes, and psychological disorders, accompanied by a lack of government support for necessities, exacerbated the needs and vulnerability of the society.

*"Unemployment and poverty increased. The government left the people with no support…" (P12).*

*"Inflation has risen much more than we expected, and life has become tough for people, and because of that, those who were not in a good financial position got worse than they used to be, that is, they stayed in rent and overnight bread … Quarantining deprived the day laborer of the opportunity to work …" (P1).*

*"Rehabilitation equipment for the disabled has become many times more expensive. How should these families provide for themselves with the low salaries they receive? Unfortunately, our legislators do not have a solution …" (P11)*

*"Our social and mental health was low, and I think Corona has multiplied. I think the situation is worrying, people in quarantine conditions who were forced to stay at home were even challenged in family interactions … " (P22).*

Due to restrictions on in-person services, reduced working hours in the NGO office, replacement of routine activities with those related to the pandemic, and Less use of volunteers due to fears of developing Covid-19, the NGOs witnessed disruption in the provision of regular services.

*"We used to have music, song, and theater talent classes with volunteers, but they were canceled. We had to reduce our working hours to be home at 9 o'clock …… Due to the law related to traffic restrictions in the city until 9 pm, we reduced the period of presence in the NGO…» (P22);*

*"About 60 to 70 percent of our goals were focused on prevention, fight and control of CORONA damages …" (P4)*

Regarding NGOs staff, the main challenge of the NGOs was the concern about the infection due to the lack of adequate protective equipment, "Right now, one person wears a mask from morning to evening, but the reasonable time to use the mask is two hours. At least 50 to 100 clients come and go in the center, and they are experiencing stressful conditions …" (P8).

*"… That is, at the beginning of the Corona crisis, we were afraid that the volunteers would get Covid 19 disease, and we used some of our volunteers less …… Now, it seems that we had a double responsibility so that one of these forces would not become like them; after all, each of them had a family …" (P8).*

Charities such as philanthropists ceased support for reasons such as being affected by the economic situation in the community and lack of interest in the NGO activity field.

*"Our charities also lost their financial means, because their business also suffered … " (P12).*

*"After the CORONA, donations from charities became very low … not that they were disconnected; they were not financially able to help. Because the economic pressure is too much …." (P10).*

Virtual communication was not always available due to the limited technology available in the country.

*"We could not attend webinars or virtual meetings. The internet speed was slow, or the mobile phone was weak, and the computers did not work …" (P11).*

*"We could not have virtual education for groups that are deprived society. They do not have smartphones; they do not have internet …” (P9).*

Loss of resources due to faulty spending and imbalances of budget and expenditures were prominent examples of inefficiency in financial resources management.

*"For example, the NGOs thought that they should provide immediate assistance to the hospitals. Without a plan and coordination, they provided many clothes, guns, and goggles for the hospitals, and all the financial resources were spent. At one point, the hospital trash was filled with the protective equipment they had sent.… Maybe it was consumed indiscriminately, maybe it was not appropriately distributed… " (P6).*

*"Some of them could not continue and ceased operation due to increased rents. Or they could not pay the salaries of the personnel and were forced to adjust their number, and on the other hand, their need for volunteer forces increased. Receipt of resources and financial aid from donors and organizations decreased…" (P2).*

### NGO challenges in interactions with government

The negative attitude of the parties to each other, Unawareness of the parties of each other's roles, plans, and capacities, Irrational expectations of the NGOs from the government, Non-recognition of the freedom of action of the NGOs, Shortcomings in the formulation and implementation of laws, Disorders in current administrative processes and Lack of support from the governing administrations were challenges of interaction with other institutions.

Some interaction problems have overshadowed and hindered the formation of optimal partnerships between NGOs and their counterparts or government. These issues have persisted throughout the Covid epidemic.

*"NGOs compete with each other in difficult economic conditions to get help from donors, and this leads to a lack of proper interaction between them. As a result, they are not informed about each other's programs. They do not use each other's capacities because they do not have good relations."(P4).*

*"An attempt was made to set up a coordination headquarters, but the coordination headquarters do not have the necessary efficiency due to the busy schedule of the managers and the lack of special EOCs, and the activities become isolated…" (P4).*

Some NGOs had unreasonable expectations for financial resource provision and profitability, which affected their relations with the government institutions.

*"… Some NGOs have their eyes on the government, which is not true, we should not go forward relying on the government …" (P1).*

*"Some people may form NGOs to make a financial profit. That is, they want to be both an NGO and earn an income and be active and contact a government agency for financial support …" (P8).*

The non-recognition and the obligation of the NGOs to move in the direction of the programs of the licensing institution was another challenge.

*"… The government does not want NGOs to present themselves, and ultimately, they want NGOs to be their executive arm. "While non-governmental organizations have the idea that if they are an arm, they are an intelligent arm and they should play a role in decision-making…" (P6).*

*".. we must act within their framework for any purpose, they do not allow us to take creative action and participate in other matters of society" (P5).*

Another part of the challenges of the NGOs occurs due to the ignorance of the other NGOs' roles, plans, and capacities.

*"The NGOs underestimate the potential for development that exists in the government … " (P6).*

*"The main activity of NGOs is for the people, but the heads of government departments do not know what NGOs do at all …" (P1).*

*Shortcomings in the formulation and implementation of laws included the lack of formulation and application of protective and restrictive government laws. "Reporting and tax collection may make it difficult to continue operation …" (P2);*

*"We will never complain about the lawlessness of the country. We are worried and upset about the non-implementation and incorrect implementation of these laws …" (P22).*

Negative attitudes mainly mean distrusting the government and the NGOs towards each other. Politicization provides the basis for this distrust, and the government's resistance to accepting the NGOs as a social movement away from ideological brands stems from it.

*"The government does not impose any special laws on the activities of the NGOs because it prefers to govern them with regulations, and it is based on distrust. The government viewed the NGOs as a Western gift and sought to make it in favor of an indigenous ideology." (P6).*

Lack of support from the ruling apparatus included tax collection, non-continuation of protections, and non-payment of previous debts.

*"During this period, we helped the officials together, they know us, and some of them are members of the institute, but they did not help. "We got a plan from the governor's office, who gave half of the money, and it is almost a year and a half, and they have not given the other half …" (P7).*

Following the outbreak, the NGOs witnessed disarray in current administrative processes due to the disruption of the chain of review of programs and laws, delays in the licensing and assistance process, and the coincidence of the crisis with the holidays.

*"In the crisis, we reached the end of the year and the offices closed. On the other hand, the first three weeks were holidays…" (P8).*

*"A common guideline was to be revised Which was interrupted during the outbreak of coronary heart disease …» (P16)*

*"The process of renewing a license by the governor takes four months, that is, you are so annoyed that there is no energy left …" (P18).*

### NGO information sources in COVID-19 crisis

Participants cited a variety of public and private organizations as sources of information, including the Ministry of Health and Medical Education and related organizations, including medical universities and health centers, the national media, the welfare organization, the municipality, especially the health department, networks, and National Committees and peers. Instructions and protocols have often been cited as rich sources of information.

*"We received instructions from the university on how much water and disinfectant to be poured to clean surfaces and hands …" (P8).*

*Dialogue and interaction between the NGOs and headquarter experts was another way to access information. "We exchanged views at the provincial level. We were asking and answering questions with the Welfare Organization about what to do…" (P11).*

*During the pandemic, the NGOs attended the training classes of government institutions, which led to an increase in their awareness. "Our staff participated in the training classes of the city health center…" (P11).*

Access to information was also facilitated through national crisis networks (e.g., COVID-19 control taskforce), provincial organizations, and the National Committee for Public Participation.

*"We had access to information through the National Crisis Management Network, the National Committee for Public Participation and the Provincial Network of Organizations …" (P4).*

*Experts also cited the results of visiting the social pages of government organizations. "An Instagram page was under the supervision of Tehran University of Medical Sciences. It was a verified page …" (P22).*

Another participant mentioned the dissemination of information by the national media. *"… In the first days, at the end of February and March, we tried to get more help from the radio…" (P11).*

#### NGO support strategies in the pandemic efforts

This category is devoted to solutions to the challenges the NGOs were exposed to during the participation process and provides the basis for continuing activities in crisis management. The government and its body are mainly responsible for implementing these strategies. Up-to-date information, planning and guidance, recognition of rights, status and role of institutions, technical and training, physical, human and financial support, promotion of a culture of social participation, and facilitating participation in decision-making were proposed solutions. The purpose of up-to-date information is to provide up-to-date scientific knowledge to the NGOs. One of the proposed measures in this field is preparing a database by scientific institutions.

*"It will be beneficial if universities and research centers provide comprehensive databases of those in need…" (P4).*

The incident command system, strategic and operational planning, and a schedule were mentioned.

*"In a crisis like in a war, there must be a single commander so that there is no parallel work … In a crisis, organizations can be divided into three general categories: specialized, semi-specialized, and general. Specialized and semi-specialized groups can be specialized in crisis management because they are trained. Public institutions have the role of training, streamlining, raising awareness, collecting and delivering aid to specialized groups …" (P4).*

Recognition of the rights, status, and role of the NGOs focuses on the commitment of governments to obtain a public opinion, trust, and grant of legal authority to these institutions.

*"The required autonomy should be given to the NGOs, and there should be rules and regulations to provide guarantees. The right to demand must be recognized so that NGOs can monitor the government's performance. The government should facilitate the process of participation of the NGOs in decision-making…" (P2).*

Technical support empowers the NGOs by providing adequate NGO staff, training in dealing with crises, and directing their actions by scientific institutions.

*"In these (crises), the NGOs had to approach the infectious disease and epidemiology specialists of the university, because in the face of this public problem, while mobilizing the public, the accurate information had to be disseminated …" (P6).*

Physical, NGO staff and financial support included reviewing guidelines, providing health items, identifying volunteers, and providing financial assistance.

*"The issue was the identification of volunteers which requires cultural promotion and awareness-raising within the community…" (P4);*

*"they should distribute masks, disposable gloves, hand gel or alcohol to give to the poor who are in the villages and cannot afford to buy …" (P12).*

Encouraging people to join and participate in community affairs is another part of the support needed by NGOs.

*"People have to practice in the field of the NGOs. This participation can be culture-building …" (P6).*

NGO involvement in decision-making is made possible by creating specialized networks and the presence of these institutions in decision-making meetings.

*"The government must provide a platform for NGO networks to enjoy a higher status in government terms. A social participation organization can share people's opinions with the government and help make decisions…" (P6).*

## Discussion

In this study, an attempt was made to investigate the activities of the NGOs in response to the COVID-19 pandemic in Iran and to identify the challenges these institutions were exposed to. Align with previous studies [1, 29–31]; our findings highlighted the necessity for NGO participation in addressing different kinds of crises such as pandemics, disasters, and conflict. In our study, Iranian NGOs involved in health primarily took preventive measures. The NGOs were effectively involved in providing mental health services. Another significant part of the NGOs' participation was activities to strengthen inter-sectoral cooperation and help improve the health care system. Mobilizing resources to support the community under coverage was another form of participation. Previous studies have also pointed to various forms of NGO involvement in response that effectively cope with COVID-19 [12–15], particularly to raise public awareness and provide services to vulnerable groups [26, 32]. In their study, Shah et al. reported the direct participation of the NGOs in the production of personal protective equipment for people who worked at the forefront of the health care system [33]. In the study of Nemțeanu et al., the activity of the NGOs during the COVID-19 outbreak was summarized in three areas of health care, care, and social and humanitarian services, respectively. In this study, other initiatives of the NGOs were the development of programs or applications for the medical staff to cope during the outbreak and the development of technologies to facilitate the access of other organizations to resources and potential stakeholders. Such efforts in the current context of the COVID-19 pandemic have highlighted the catalytic role of NGOs in bridging the gap between government and the people [34]. Given the role of NGOs during global challenges, investing in their capacity building is inevitable.

Depending on the circumstances of each country and the upstream laws, the nature of NGO participation differed within a range of minimum to maximum. Assistance in providing health training services, supporting vulnerable groups, and establishing communication between the government and the people have been the most common forms of NGO involvement [35]. However, it is suggested that to create unity of procedure and reduce ambiguity in playing the role, the authority and responsibilities of the NGOs in the field of health to be codified and informed so that both every day and critical situations can be used more efficiently to improve performance and accelerate the reaction.

According to the present study's findings, accessing and serving the target groups was one of the challenges that the NGOs were exposed to in their activities during the COVID-19 pandemic. The target community of many NGOs active in health includes vulnerable individuals and identification, gaining trust, establishing constructive relationships, and encouraging them to receive efficient services provided by the NGOs requires special skills and is not easily possible. This complexity has been exacerbated in times of crisis, especially during COVID-19, which has affected all aspects of the community's life.

Another challenge identified in the study was the interaction of the NGOs with governmental and non-governmental institutions. Lack or weak constructive cooperation and exchange of the NGOs with the government or other institutions is one of the common problems mentioned in other studies [36, 37]. Insufficient knowledge on NGOs and their role in assisting the health system and poor governance of the health sector for transparency and accountability to determining the role of health stakeholders are the main reasons for the lack of constructive interaction between the NGOs and other institutions. In emergencies, such a defect becomes more apparent. It can lead to more confusion in the continuity of health services due to inconsistency and parallelism and further the inefficiency of the health system [38–40].

A review of previous studies has reported other challenges for NGO activities during the COVID-19 pandemic. For example, a survey by Wilke et al. in July 2020 showed that NGOs experienced government restrictions, reduced funding, and the inability to provide services during the COVID-19 outbreak [17]. The results of the Deilamizadeh study also showed that since the outbreak, NGOs involved with the protection of the homeless and addicts against COVID-19 were exposed to challenges such as health problems related to drug use and distribution sites, isolation and care, and training problems due to lack of access to smartphones to receive the necessary training programs. Problems associated with the reduced staff support and, the possibility of increased burnout after the COVID-19 pandemic, problems with the lack of supply of disinfectants and medical equipment have been other challenges that NGOs have been exposed to in this [41]. A closer look at the list of different problems expressed by the NGOs during the crisis indicates that the most critical problems are the NGOs' access to the target groups and the lack of a communication network and constructive interaction between government institutions and the NGOs.

Another part of the study findings identified solutions to address the NGOs' challenges in response to COVID-19. These strategies mainly focused on recognizing the role and position of the NGOs, systematizing and organizing their responsibilities, authority, and relations, and providing more technical support and assistance. Considering that the NGO challenges vary in different environments, diverse solutions have been proposed in previous studies to address them. For example, in the DíAZ study, considering the problems that occurred during the COVID-19 pandemic for a non-governmental organization in the United States, three recommendations were made to help the populations to respond quickly to the situation: more focus on internal and external actors of the organization, identification of organizational capabilities to cope and more emphasis on accountability and transparency [42]. Cheng et al., citing the excellent performance of Zhejiang Province in controlling COVID-19, discussed the role of community-based organizations in this success, and, based on their findings, recommended that governments should use the strengths of community-based organizations in responding to COVID-19; encouraging volunteer participation in pandemic prevention and control; provision of data infrastructure and digital tracking system and built of long-term trust and capacity in community-based organizations [16]. Maserat et al. also point to the role of a web portal in facilitating collaboration between NGOs and academia during the COVID-19 outbreak and suggest a severe need for web portal capabilities to control COVID-19 in the digital age. Also, the cooperation of training and academic institutions in health portals can play an important role in covering the COVID-19 pandemic. The interactive portal is one of the advanced technologies NGOs can use for health management [43].

There are reports in Iran suggesting that some of these strategies were considered during the COVID-19, and to some extent, the obstacles to government cooperation with the NGOs were resolved. According to these reports, people have participated in the response process through associations or groups, such as religious and ethnic communities and non-governmental organizations [44]. Despite this evidence, according to the present study's findings, there is still a need to improve and enhance the participation of NGOs. Considering the recommended strategies to promote NGO participation in mapping to respond to COVID-19 and also the main current challenges the active NGOs in the field of health are exposed to during the pandemic, it is proposed to design national programs to empower the NGOs to identify and access the target population and conduct interventions to form and institutionalize the status of the NGOs and their communication network with the public sector.

## Limitations of the study

The study was conducted among managers of health-related NGOs. Although the context in which NGOs operate is the same, the findings of this study may not apply to other NGOs. Second, due to COVID-19 restrictions, we conducted the interviews in absentia, which may not have created a comfortable space for discussion. Further studies are recommended to perform with more representatives of NGOs with quantitative approaches and reviews to examine the challenges and find practical interventions to address them.

## Conclusions

The critical role of NGOs in assisting the government in crises management necessitates their empowerment for better response. This necessity was even more significant after the COVID-19 crisis and the experiences that demonstrated the effective involvement of the NGOs in coping with this crisis. However, it is suggested that this capacity be used more effectively by codifying and defining the authorities and responsibilities of NGOs in critical situations. Also, considering the crucial challenges for their participation, such as the NGO access to the target groups, lack of communication network, and constructive interaction between government institutions and the NGOs, it is recommended to increase the capacity of these institutions and intervene to establish a constructive and long-term relationship with the government.

## Data Availability

The datasets used and/or analyzed during the current study are available from the corresponding author on reasonable request.

## Abbreviations

NGO: Non-Governmental Organizations
